# GuiTope: an application for mapping random-sequence peptides to protein sequences

**DOI:** 10.1186/1471-2105-13-1

**Published:** 2012-01-03

**Authors:** Rebecca F Halperin, Phillip Stafford, Jack S Emery, Krupa Arun Navalkar, Stephen Albert Johnston

**Affiliations:** 1 Center for Innovations in Medicine, The Biodesign Institute at Arizona State University, PO Box 875901, Tempe AZ 85281, USA

## Abstract

**Background:**

Random-sequence peptide libraries are a commonly used tool to identify novel ligands for binding antibodies, other proteins, and small molecules. It is often of interest to compare the selected peptide sequences to the natural protein binding partners to infer the exact binding site or the importance of particular residues. The ability to search a set of sequences for similarity to a set of peptides may sometimes enable the prediction of an antibody epitope or a novel binding partner. We have developed a software application designed specifically for this task.

**Results:**

GuiTope provides a graphical user interface for aligning peptide sequences to protein sequences. All alignment parameters are accessible to the user including the ability to specify the amino acid frequency in the peptide library; these frequencies often differ significantly from those assumed by popular alignment programs. It also includes a novel feature to align di-peptide inversions, which we have found improves the accuracy of antibody epitope prediction from peptide microarray data and shows utility in analyzing phage display datasets. Finally, GuiTope can randomly select peptides from a given library to estimate a null distribution of scores and calculate statistical significance.

**Conclusions:**

GuiTope provides a convenient method for comparing selected peptide sequences to protein sequences, including flexible alignment parameters, novel alignment features, ability to search a database, and statistical significance of results. The software is available as an executable (for PC) at http://www.immunosignature.com/software and ongoing updates and source code will be available at sourceforge.net.

## Background

Random-sequence peptide library screening approaches represent an increasingly popular and powerful tool for identifying binding partners for antibodies and other proteins as well as carbohydrates, pharmaceuticals, and other small molecules. Peptide library methods generally fall into two categories: molecular display approaches such as phage display, and immobilized arrays such as SPOT. Display approaches can typically accommodate much larger libraries, but information is typically obtained only on the clones that survive several rounds of panning, resulting in a population that is heavily biased in favor of clones whose sequences facilitate growth [1]. In contrast, array based approaches may be used to screen smaller libraries with higher throughput than display approaches and semi-quantitative binding information is obtained on all of the peptides in the library. New technologies both on the display side and the array approach promise to overcome these limitations [2-4]. The decreasing cost of both sequencing and peptide synthesis as well as applications such as profiling the humoral immune response [5] promise to increase interest in connecting random-sequence peptide mimotopes to protein sequences occurring in nature. Therefore, an increase in the demand for appropriate algorithms and software to facilitate the data analysis would also be expected.

While the peptides discovered in these library screening experiments serve as useful ligands in and of themselves, comparison of these sequences to natural protein sequences can reveal novel biological insight. Peptides selected by panning phage display libraries against monoclonal antibodies often closely match the antibody epitope making the sequence comparison rather straightforward [6]. If a strong enough motif is uncovered among the peptide sequences, it may even be used to search a database to predict an antibody target [7]. Though current array technology does not allow sufficient coverage of sequence space to contain sequences closely resembling natural protein sequences by chance, we have shown that experiments of this type still have utility for predicting monoclonal epitopes [8]. Other groups have shown that peptides selected to bind to other types of proteins have utility in understanding and predicting binding to natural binding partners [9-11]. Even small molecule binding peptides provide insight on their binding to natural proteins [3,12].

Analysis of the peptide sequences obtained from any selection experiment poses two key challenges. First, a set of peptides need to be compared against a protein database. Second, an appropriate scoring scheme is needed to search for structural similarity rather than evolutionary relationships. At first glance, the FASTS/FASTF programs appear to address the first challenge, as they are designed to take peptide sequences generated from protein sequencing techniques and identify homologous proteins [13]. However, the FASTS/FASTF programs search for cases where peptides align to non-overlapping regions of the protein sequence, while we would like to identify regions where the peptides align to the same region of the protein sequence. Another approach is to identify a motif among the selected peptide sequences and use the consensus sequence or a probabilistic representation of the motif to compare to the protein sequence(s) of interest [14]. We previously demonstrated that the glam2 motif finding program is suitable for analyzing random-sequence peptide data [8,15]. While the motif approach may be powerful in many cases, the peptides of interest may not always have a common pattern because different amino acids may match in the same region of the sequence, or peptides may align to different parts of the protein sequence(s). Another approach would be to align each discovered peptide sequence to the protein sequence targets and sum the alignment scores at each position. The RELIC MATCH program (not currently available or supported) used this approach with some success [3,9,10,12,16]. This program also had several limitations with regards to transparency, flexibility, statistical analysis, and the ability to search multiple sequences. Here we present an open source application that gives the user access to all parameters, can empirically estimate the statistical significance of the results, and enables the analysis of many sequences at once.

## Methods

### Algorithm overview

The user inputs protein sequence(s) to search, a set of selected peptides, and (optionally) a representative or complete list of peptides from the library. A scoring matrix may be generated by the program as described below or entered by the user. The maximal local alignment between each selected peptide and protein sequence is found. If the alignment score is greater than the user defined score threshold, the score at each protein residue position is added to the protein residue scores. If the moving average window size is set to greater than one, after all peptides have been aligned to a given protein, the moving average across the protein residue positions is calculated and the residue scores provided correspond to the score at the start of the window. The same number of peptides as in the selected list are randomly selected from the library if a library set was entered, and these are aligned to the protein(s) in the same manner as for the selected peptides; this process is repeated for the specified number of sampling iterations. If the subtract library scores box is checked, the average scores at each residue position from the randomly selected peptides from the library are subtracted from the residue scores. The selected peptide scores across each protein sequence are graphed, as well as the maximum and average scores from the random sampling iterations. The user may use the sort button to order the proteins by their maximal residue scores. The text output tab may be used to view a summary table of the maximum alignment scores for each protein or a table of all of the alignments identified for the number of proteins specified.

### Scoring matrix

GuiTope generates a log-odds-like scoring matrix based on a given measure of amino acid distances and amino acid frequencies. The distance matrix is taken to be inversely proportional to the frequencies of an amino acid pair appearing in a true alignment after a pseudocount of 10% of the average distance is added to the distance matrix to avoid dividing by zero. The rows and columns are iteratively scaled to sum to the expected amino acid frequencies. This matrix is then divided by the product of protein and peptide amino acid frequencies at each position and log_10 _transformed.

### Alignment algorithm and inversion scoring

The maximal gapless local alignment of each peptide with each protein is calculated using the Smith-Waterman algorithm. If the inversion weight is set to greater than 0, the program will identify sequence positions where the protein residue at position i is the same as the peptide residue at position j +1 AND the protein residue at position i+1 is the same as the peptide residue at position j. The residue scores for these inversions will be the product of the inversion weight and the average of the identity scores for the amino acids at the protein positions i and i+1.

### Statistical analysis

For each sampling iteration and each protein sequence, a set of peptides, with the same number of peptides as the selected peptide list, is randomly selected from the library and the residue scores are calculated. From these, the maximum and average residue scores are calculated for each position. If the 'subtract library scores' option is selected, the average library scores are subtracted from the residue scores from each iteration. The maximum scores from each protein iteration are ranked. For each protein, the maximum residue score from the selected peptides is compared to the ranked scores. The percentage of library scores that are higher than the selected peptide score is reported as the significance.

### Evaluation datasets

A dataset was previously described containing lists of peptide sequences identified from random-sequence peptide microarray experiments as binding to monoclonal antibodies with known epitopes [8]. This dataset was used to optimize Guitope's alignment parameters. A polyclonal anti-peptide dataset from the same publication was used to evaluate the algorithm. Additionally, another set of monoclonal antibodies with known epitopes was used to probe a completely different set of 10,000 random-sequence peptides on a microarray. The two anti-P53 antibodies from the first monoclonal antibody dataset were repeated on both the first and second version of the 10,000 peptide microarrays. Additionally, an anti-cMyc clone 9E10 (AbD SeroTec, Raleigh), anti-Leu-Enkaphalin clone 1193/220 (AbD SeroTec, Raleigh), anti-PBEF clone E10 (Santa Cruz Biotechnology), and anti-V5 (AbD SeroTec, Raleigh) were used to probe the array and generate lists of peptides to which the antibodies bound. Anti-cMyc, anti-Leu-Enkaphalin, and anti-V5 recognize epitope tags, while the anti-PBEF was epitope mapped using tiling peptides (current authors, manuscript in preparation). Phage display datasets that identified the greatest number of unique peptides were selected from those listed in the "several binding sites" category in Derda *et al*. [1] and these were downloaded from MimoDB http://immunet.cn/mimodb/. These phage display datasets include peptides selected against a diverse set of targets, including two human extracellular proteins, one bacterial protein, and immune sera to a virus and a bacterium.

### Implementation

GuiTope was implemented in Visual Basic, using the Microsoft .NET framework. It may be installed on any computer running Microsoft Windows XP or a newer Windows operating system. It has a memory footprint of 400 MB and will take anywhere between seconds to several minutes to run a set of hundreds of peptides against a single protein with 100 sampling iterations on a single Pentium 4 core, 3.2 GHz and 2 GB RAM machine running Windows XP. On the same hardware, searching a protein database of ~20,000 proteins with a set of several hundred peptides with a single sampling iteration, will utilize < 3 GB of memory and use approximately 20 hours of direct CPU time.

## Results and discussion

**Figure 1:**
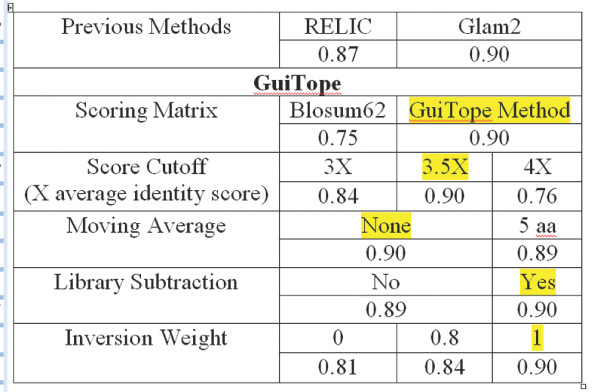
**Parameter Optimization**. The AUROC (Area Under the Receiver Operator Characteristics Curve) is shown for each parameter value tested on the 1^st ^Known Epitope Monoclonal Dataset shown below. The best parameter value was highlighted and that value was used when each other value was varied.

The optimal combination of parameters for GuiTope was determined by testing on a previously described dataset of peptide sequences bound by monoclonal antibodies with known epitopes that had been used to probe a random-sequence peptide array. [8] Epitope predictions were evaluated using ROC analysis and the AUROC scores are reported in Figure 1. The most critical parameter appears to be the scoring matrix, with the BLOSUM62 matrix having an AUROC 0.15 less than the GuiTope method which adjusts for altered amino acid frequencies. The di-peptide inversion method also had a substantial improvement in the AUROC score. The di-peptide inversion method is a novel alignment approach that we developed after observing such alignments in our data. We hypothesize that the flexibility of the peptides enables the inverted amino acids to have similar interactions with the paratope and we have found some preliminary experimental and modeling evidence supporting the di-peptide inversion (data not shown). Here we have included results from analysis with and without di-peptide inversions since the approach is unusual. The library subtraction method only yields a small improvement to the score and a large number sampling iterations are required to accurately estimate the average library score, so we only used library subtraction for evaluating individual proteins rather than for database searches in order to keep run times reasonable.

**Figure 2:**
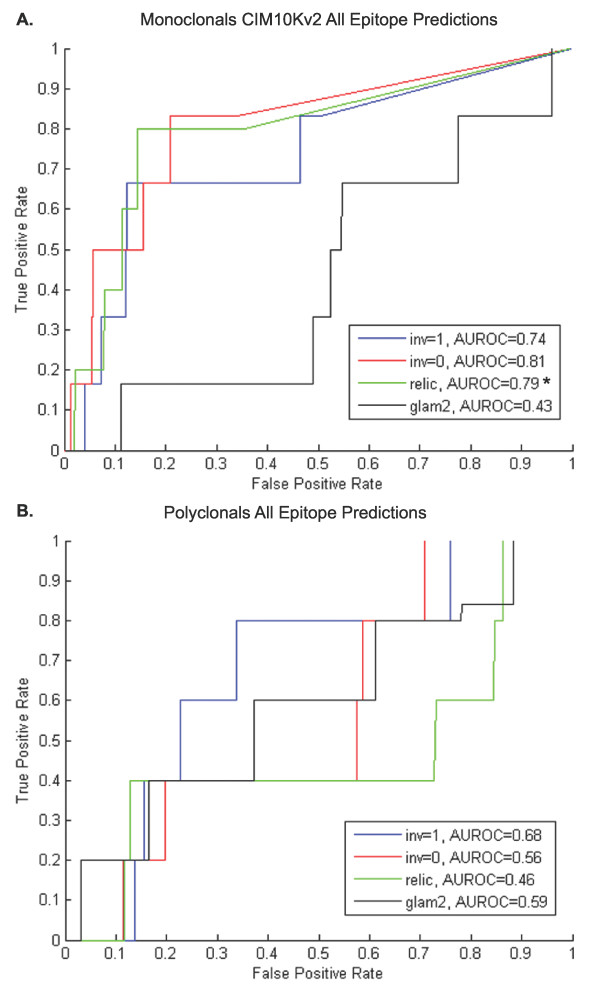
**Peptide Microarray Evaluation Datasets**. Peptides selected to bind known epitope monoclonals (A) or anti-peptide polyclonal sera (B) were used to predict the epitope (A) or immunizing peptide (B) in GuiTope, RELIC, or Glam2 within a database of decoy sequences. The significance scores of the true epitope or immunizing peptide sequences was compared to the decoy sequences using ROC plots, where the true positive rate is plotted against the false positive rate for all possible score thresholds. The results using the inversion weight as one are plotted in blue, the results without inversions are plotted in red, the results for RELIC are plotted in green, and the results for Glam2 are plotted in black. The AUROC value shown in the legend indicates the probability that a true sequence would score higher than a decoy sequence for that dataset. *Note that the RELIC analysis of the monoclonal set is only based on five monoclonal antibodies because the sixth antibody was run after the server was no longer available.

GuiTope was tested on two independent datasets obtained by probing random-sequence peptide microarrays with antibodies. The first was obtained by probing an array of 10,000 random-sequence peptides, having completely different sequences than those used in the training set, with monoclonal antibodies having known linear epitopes. The monoclonal epitopes were predicted with an AUROC score of 0.75 using the inversion method and 0.78 without inversions (Figure 2A). It appears that this dataset is more difficult to predict as the RELIC and glam2 methods also perform worse. The second peptide microarray evaluation dataset was generated from polyclonal anti-peptide sera. Here GuiTope performs similarly to previously tested methods with an AUROC of 0.68 using the inversion method and 0.56 without inversions, compared to an AUROC of 0.48 using RELIC method and 0.68 using Glam2 (Figure 2B). These microarray datasets are likely considerably more difficult than phage display datasets because of sparse sampling of sequence space.

**Figure 3:**
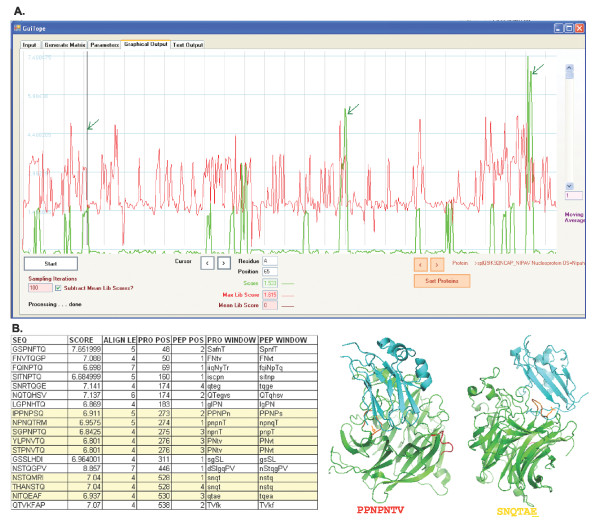
**Analysis of Anti-Nipah Dataset**. A. Screen shot of GuiTope mapping of anti-Nipah Virus selected peptides to the Nipah Nucleoprotein. Epitopes previously predicted and validated from this phage display peptide set are indicated with arrows. B. Novel GuiTope predictions using the inversion method of Nipah Glycoprotein G epitopes. The GuiTope alignment detail is shown as well as the locations of these epitopes in the crystal structure. The underlined glutamic acid is part of the receptor binding site.

Phage display datasets evaluated in GuiTope were selected based on the summary of the MimoDB published in Derda *et al*. [1]. Two of these datasets consisted of peptides selected to polyclonal sera. The phage display peptides selected against the anti-Nipah virus were used to map three epitopes on the nucleoprotein, and GuiTope also identified these epitope regions (Figure 3A). GuiTope also predicted an epitope on Glycoprotein G that was also predicted by DiscoTope [17], which uses the crystal structure to identify accessible regions (Figure 3B). Yang *et al*. identified some regions of sequence similarity between the anti-*Mycoplasma hyopneumoniae *selected peptides and several *M. hyopneumoniae *protein sequences, but did not test whether their epitope predictions were correct [18]. The only experimentally determined *Mycoplasma hyopneumoniae *B-cell epitopes in the Immune Epitope Database [19] were determined by a peptide tiling study of predicted lipoproteins [20]. None of these epitopes were predicted by the Yang *et al*. or the GuiTope analysis. Most likely the phage display selected peptides correspond to epitopes on proteins other than the lipoproteins. There is no structural or experimental data to evaluate GuiTope's predictions of the *Mycoplasma hyopneumoniae *epitopes.

**Figure 4:**
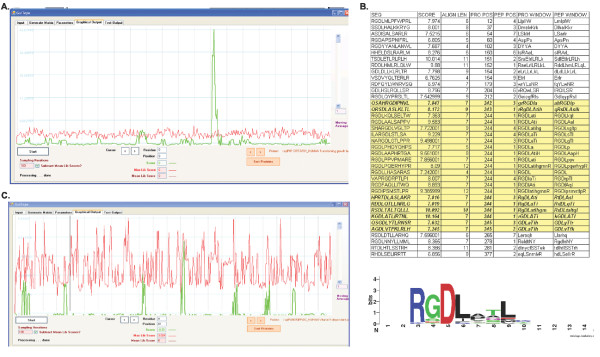
**Protein Interaction Predictions**. A. Peptides selected to bind Integrin AlphaV Beta6 clearly aligned in GuiTope to the integrin binding site on TGF beta 1. B. Detailed alignments of the peptides to TGF beta 1, with those that align to the binding site highlighted in yellow and those that do not contain the RGB motif shown in italic. Below the WebLogo view of peptides aligning to the region illustrates the relative importance of amino acids C. Peptides selected to bind to EPCR do not align to a particular region on protein C.

Three protein panning datasets were also evaluated. In the first example, White *et al*. did not identify any similarity between the peptides found to bind to the endothelial protein C receptor (EPCR) and Protein C or any other known EPCR binding partners. GuiTope likewise did not find any significant similarity between any known EPCR interactors (Figure 4C). In the second case, the peptides selected to bind to integrin α5β6 were mapped by GuiTope to the known interactors TGF beta 1 and TGF beta 3 as two of the top three hits (Table 1) and Guitope correctly identified the important interacting amino acids (Figure 4B). Since these interactions were discovered after the publication of the phage display study, one may suppose that they could have been predicted from the phage display data if the proper analysis tools had been available. In the third set, neither Carettoni *et al*. nor the Guitope analysis reveal a clear similarity between the FtsA binding peptides and a known FtsA interactor. Carettoni *et al*. identified a weak motif that matched a site on FtsA, and used that site to develop a model for the structure of the FtsA dimer [21]. While several lines of evidence suggest that *E. Coli *FtsA does form a dimer, it is not clear whether the model proposed based on this phage-display data is correct [22]. We are not aware of any experimental evidence to support or refute the interactions predicted by GuiTope.

**Table 1:** Phage display database search

MimoDB/Reference	Target	Database (number of Proteins)	Known Interactor	Rank, p-value (Inv/No Inv)
288 [24]	Endothelial protein C receptor	Human Extracellular and Cell Surface Proteins (5074)	Protein C	NA*
148 [25]	Polyclonal Anti-Nipah Virus	Nipah Proteome (9)	Nucleoprotein	2, < 0.1/ 2, < 0.1
753,754,755 [26]	Integerin α5β6	Human Extracellular and Cell Surface Proteins (5074)	TGF beta 1 TGF beta 3	2, < 0.0002/ 1, < 0.0002 3, < 0.0002/ 2, < 0.0002
204-205 [18]	Anti-*M. hyopneumoniae *polyclonal antibody	*Mycoplasma hyopneumoniae *Proteome (691)	Lipoproteins and p97	None matched correct region
1127 [21]	*Escherichia coli *FtsA	*Escherichia coli *(4311)	FtsA	1106,0.25/ 2067,0.58

*Protein C was not listed as Extracellular or a Cell Surface Protein GO annotation

The peptides that bind to a given target do not always have sequences that are similar to biologically relevant proteins. This problem is confounded when peptide array approaches are used because peptides that are highly similar to a given protein are unlikely to be present in the library. GuiTope was able to take these loosely similar sequences and predict antibody epitopes with modest accuracy (AUROC 0.75-0.9) in line with previously tested methods [8]. Random-sequence peptide microarrays have shown great promise in profiling the humoral immune response [5,23], and it would be of great utility to be able to use the peptide sequences to trace back to the antigen that elicited the immune response. However, the current prediction accuracy would not be sufficient for this task [8]. In contrast to the peptide array datasets, the phage display selected peptides can sometimes be used to predict interaction partners from a database very accurately. As less biased molecular display methods are developed and higher density peptide arrays become available, we expect that the information content of the peptide sequences will improve, making the type of analysis facilitated by GuiTope even more useful.

### Availability and requirements

The executable is available on http://www.immunosignature.com/software  and will install and run on any PC with Windows XP or later. The source code is written in Visual Basic and available on sourceforge.net. The Microsoft .NET framework is required.

## Authors' contributions

SAJ conceived the project. JE and RFH designed and implemented the software. KAN suggested the di-peptide inversion method. KAN and PS tested the software. RFH, PS, and SAJ designed the evaluation strategy. RFH performed the evaluation and drafted the manuscript. PS, SAJ, and KAN critically revised the manuscript. All authors have read and approved the final version of the manuscript.
